# Pain assessment during physiotherapy and noxious stimuli in patients with disorders of consciousness: A preliminary study

**DOI:** 10.3389/fnint.2022.962077

**Published:** 2022-09-08

**Authors:** Jianzhong Shen, Shanchun Tang, Bingyang Yan, Donghua Xie, Tingting Fang, Lidan Chen, Guoyun Li

**Affiliations:** Shanghai Yongci Rehabilitation Hospital, Shanghai, China

**Keywords:** nociception coma scale-revised, disorders of consciousness, minimally conscious state, physiotherapy, pain

## Abstract

**Objectives:**

The primary purpose of this study is to determine whether patients with disorders of consciousness (DOC) (unresponsive wakefulness syndrome, UWS; minimally conscious state, MCS) experience pain during physiotherapy and noxious stimuli in a larger patient population.

**Materials and methods:**

The patients’ level of consciousness was measured with the Coma Recovery Scale-Revised (CRS-R). Additionally, the Nociception Coma Scale-revised (NCS-R) was used to assess their pain response. The NCS-R total scores between UWS and MCS at baseline, physiotherapy and noxious stimulus were compared using the Mann-Whitney U test (Wilcoxon rank-sum test) and the Kruskal-Wallis H test with Bonferroni correction.

**Results:**

The study enrolled 93 participants. There was a statistically significant difference in NCS-R total scores between the three conditions (*H* = 215.25, *p* < 0.001). At baseline, there was no statistically significant difference between MCS and UWS (U = 378, z = –1.35, *p* = 0.178). While there was a statistically significant difference between MCS and UWS during physiotherapy (U = 1,362, z = –3.06, *p* < 0.01) and under noxious stimuli (U = 5142.5, z = –11.22, *p* < 0.001).

**Conclusion:**

Physiotherapy improved the activity responsiveness of DOC patients, and patients experienced less potential pain. However, some DOC patients, especially MCS patients, perceived pain under the noxious stimuli.

## Introduction

Pain and nociception are critical to quality of life and survival and tend to be significant health problems (Primm et al., 2004; Baliki and Apkarian, 2015). Nociception is the neurological process of encoding and transmitting noxious stimuli, whereby signals from nociceptors in the peripheral nervous systems are transmitted to the central nervous system (Loeser and Treede, 2008). Nociceptors are sensory receptors in the skin, muscles, bones, and internal organs that detect potentially harmful stimuli. Pain is an unpleasant physical sensation or emotion expressed verbally, vocally, or behaviorally through deadpans, sobbing, or agitation that often begins with the activation of these nociceptors (Gold and Gebhart, 2010; Brown et al., 2018).

Disorders of consciousness (DOC) (Bernat, 2009; Giacino et al., 2018) mainly include unresponsive wakefulness syndrome (UWS) (Laureys et al., 2010; Monti et al., 2010) and minimally conscious state (MCS) (Giacino et al., 2002; Bruno et al., 2011). The patient will have emerged from a minimally conscious state (EMCS) when he or she demonstrates functional communication or functional use of objects (Nakase-Richardson et al., 2008; Machado et al., 2010). Generally, pain is recognized only when the patient subjectively reports it (Merskey and Bogdduk, 1986). However, clinicians and caregivers often underestimate pain in some patients, particularly those with cognitive impairment and communication difficulties (Snow et al., 2004). This can lead to clinically ineffective pain management and incorrect mediation interventions, which adversely affects the quality of life of DOC patients. Therefore, clinical management of DOC patients requires a reliable diagnosis of level of consciousness and a sensitive pain assessment tool. Studies have shown that the Nociception Coma Scale (NCS) and its revised version (NCS-R) (Schnakers et al., 2010; Chatelle et al., 2012) are now more commonly used to assess pain response in DOC patients (Chatelle et al., 2013; Sattin et al., 2013; Mcnett, 2016; Vink et al., 2017).

Studies in healthy subjects reveled that a noxious sensory stimulus triggers neuronal activity in an extensive network of brain areas, including the anterior part of the cingulate cortex (ACC), the primary and secondary somatosensory cortex, and the insula, which is involved in sensory and cognitive-affective processing and nociception (Apkarian et al., 2005; Duerden and Albanese, 2013; Ong et al., 2019). The strength and magnitude of brain responses elicited by noxious stimuli may be related to the extent of pain perception (Apkarian et al., 2005; Wager et al., 2013). Imaging studies of DOC patients have shown that almost all MCS patients and a few UWS patients have significant ACC activation in pain and emotion processing following a noxious stimulus (Laureys et al., 2002; Coghill et al., 2003; Moisset and Bouhassira, 2007; Boly et al., 2008; Chatelle et al., 2013; Markl et al., 2013; Calabrò et al., 2021).

Disorders of consciousness patients in the subacute phase are medically stable; therefore, more attention is often paid to the assessment of pain when there is evidence of pain. From previous studies, increased spasticity and autonomic nerve activity may occur in patients with brain injury (Kyle and McNeil, 2014; Bartolo et al., 2016). The following causes of pain may be common in DOC patients (Barr et al., 2013; Zasler et al., 2021, 2022): spasticity (Thibaut et al., 2015), ossification, dystonia, rigidity, contractures, musculoskeletal pain, neuropathic pain, shoulder subluxation, scoliosis, complex regional pain syndrome, and/or pressure ulcers, which are more likely to cause potential pain during care and mobilization. According to a previous case report on pain management (Lanzillo et al., 2016), relieving the pain of DOC patients with spasticity may improve the diagnosis of the patient’s consciousness. At the same time, however, excessive analgesia can lead to increased drowsiness and decreased wakefulness in patients, which can lead to misdiagnosis and even affect prognosis. Another case report of improvement in cognitive responses after continuous intrathecal baclofen treatment for spasticity (and secondarily for pain) pointed to spasticity as a factor that should be better evaluated as a possible source of spontaneous pain and evoked/exacerbated pain during physiotherapy (Formisano et al., 2019). Therefore, accurate and timely assessment of patients’ pain response during physiotherapy is critical for diagnosis, pain management, rehabilitation of movement function, and recovery of consciousness. A previous study with a small sample size on this aspect found that 83.3% (15/18) of patients showed potential pain during physiotherapy (Bonin et al., 2022).

The purpose of this study was to investigate whether DOC patients experience pain during physiotherapy in order to gain preliminary behavioral evidence for pain management in movement function rehabilitation in a larger patient population.

## Materials and methods

### Participants

Patients were recruited from the neurological rehabilitation center of Shanghai Yongci Rehabilitation Hospital (Shanghai, China).

The inclusion criteria were as follows: (1) at least 16 years of age, (2) acquired brain injury, (3) time since injury ≥ 28 days, and (4) no use of neuromuscular blockers or sedatives within 72 h before enrollment. Exclusion criteria: (1) functional impairment due to progressive mental illness, (2) persistent seizures, (3) unstable vital signs, (4) comatose, and (5) double upper-limb frustration, or fracture.

This study protocol was approved by the Ethics Committee of Shanghai Yongci Rehabilitation Hospital, which complies with the Code of Ethics of the World Medical Association (Declaration of Helsinki). Written informed consent was also obtained from the guardians relatives of the patients who participated in this study.

### Study procedure

All included patients were repeatedly assessed during hospitalization by trained professionals using the JFK-Coma Recovery Scale-Revised (CRS-R) (Giacino et al., 2004; Wannez et al., 2017). Patients’ diagnoses were primarily determined by the best diagnosis on the repeated CRS-R assessment. The NCS-R was used to assess pain response in all patients at least twice. To determine the influence of consciousness on pain response, an NCS-R assessment was performed approximately half an hour after the CRS-R assessment. The highest assessment value is the final NCS-R value after multiple assessments. Given the possible influence of circadian rhythm on assessment results, NCS-R assessments were performed in the morning and/or afternoon of hospitalization (Cortese et al., 2015). The standard NCS-R consists of three subscales that assessing motor, verbal and facial responses, with subscale scores ranging from 0 to 3.

The detailed NCS-R assessment procedure for each assessment in the current study is described below: At baseline, no stimuli were administered to the patient, and only the spontaneous motor, verbal, and facial responses were assessed with the NCS-R; physiotherapy was performed by the physical therapist, and then the patient’s behavioral responses were recorded during therapy; the noxious stimulus (i.e., the standard NCS-R, pressure on the nail bed of the right and left hands) was administered by the rater, and the patient’s behavioral responses were recorded with the NCS-R during the 10 s after each occurrence of the noxious stimulus.

In general, DOC hospitalized patients received 20 min of physical therapy (manual therapy) 5 days per week, consisting primarily of muscle manipulation, joint mobilization, and joint manipulation. The goal of therapy was to relieve pain, increase range of motion (ROM), reduce or eliminate soft tissue inflammation, improve contractile and non-contractile tissue repair, stretch, and/or stability, facilitate movement, and improve function.

### Statistical analysis

Descriptive statistics were evaluated for all demographic information. Numbers, percentages, medians, and interquartile ranges (IQR) were generated for categorical variables, while means and standard deviations (SD) were calculated for the CRS-R and NCS-R scores.

The Mann–Whitney U test (Wilcoxon rank-sum test) was used to determine whether there were differences in NCS-R values between MCS and UWS at baseline; the same procedure was used under conditions of physiotherapy and noxious stimuli. The Kruskal-Wallis H test with Bonferroni correction was used to compare NCS-R values in MCS patients at baseline, physiotherapy, and noxious stimuli conditions to determine if there were significant differences. Statistical significance was considered, and all statistical tests were two-sided (*p* < 0.05). All operations were performed using the Statistical Package for Social Sciences (SPSS) version 20.0.

## Results

This study enrolled 93 participants (60 MCS and 33 UWS). The CRS-R total scores ranged from 3 to 16 (mean ± SD: 8.56 ± 3.25) for all DOC patients; from 6 to 16 (mean ± SD: 10.28 ± 2.6) for MCS patients; and from 3 to 10 (mean ± SD: 5.42 ± 1.48) for UWS patients. In addition, Table 1 lists the characteristics (numbers, percentages, medians, and IQR or mean ± SD) and total NCS-R scores of all included research participants. The NCS-R scores of all patients are shown in the Supplementary Table 1.

**TABLE 1:** Demographic characteristics and clinical data of disorders of consciousness (DOC) patients (*n* = 93).

	Whole group (*n* = 93)	MCS (*n* = 60)	UWS (*n* = 33)
**Sex, *n* (%)**			
Male	71 (76.3)	45 (75.0)	26 (78.8)
Female	22 (23.7)	15 (25.0)	7 (21.2)
**Etiology, *n* (%)**			
TBI	47 (50.5)	31 (51.7)	16 (48.5)
NTBI	46 (49.5)	29 (48.3)	17 (51.5)
Age range at onset, y	20–75	20–75	27–73
Mean (SD) age at onset, y	51.73 (13.8)	50.77 (14.04)	53.48 (13.39)
Median time of post-injury (IQR), m	4 (1–22)	4 (1–22)	4 (1–12)
Mean (SD) time of post-injury, m	5.24 (4.02)	5.44 (4.48)	4.86 (3.03)
Median scores of CRS-R (IQR)	8 (3–16)	9.5 (6–16)	5 (3–10)
Mean (SD) scores of CRS-R	8.56 (3.25)	10.28 (2.6)	5.42 (1.48)
**Median scores of NCS-R (IQR)**			
Baseline	1 (0–2)	1 (0–2)	1 (0–2)
Physiotherapy	1 (1–3)	1 (1–3)	1 (1–2)
Noxious	4 (2–8)	5 (3–8)	3 (3–5)
**Mean (SD) scores of NCS-R**			
Baseline	0.68 (0.59)	0.7 (0.56)	0.64 (0.65)
Physiotherapy	1.45 (0.63)	1.55 (0.7)	1.27 (0.45)
Noxious	4.38 (1.28)	4.87 (1.27)	3.48 (0.67)

IQR, interquartile range; SD, standard deviation; DOC, disorders of consciousness; MCS, minimally conscious state; UWS, unresponsive wakefulness syndrome; TBI, traumatic brain injury; NTBI, non-traumatic brain injury; CRS-R, coma recovery scale-revised; NCS-R, nociception coma scale-revised.

When comparing the NCS-R total scores of all DOC patients between conditions, there were statistically significant differences in NCS-R total scores between the three conditions (H = 215.25, *p* < 0.001), including a significant difference between physiotherapy and baseline (*p* < 0.001), a significant difference between noxious stimuli and baseline (*p* < 0.001), and a significant difference between noxious stimuli and physiotherapy (*p* < 0.001).

**FIGURE 1:**
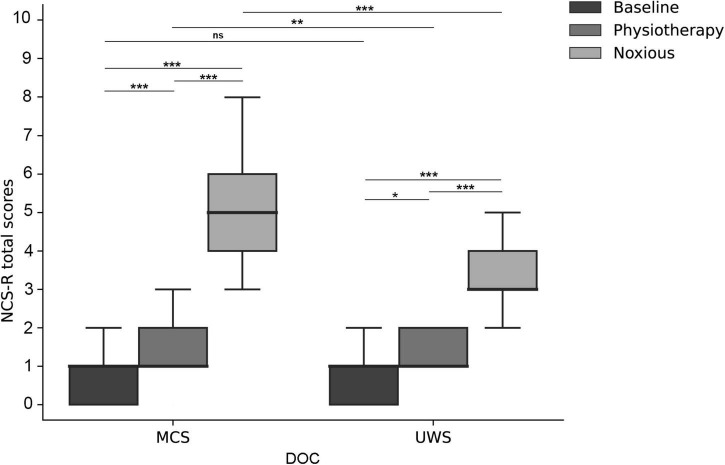
Boxplot, patients’ NCS-R total scores with different stimuli (i.e., baseline, physiotherapy, and noxious stimulus) for all DOC patients, MCS patients, and UWS patients. *p < 0.05; **p < 0.01; ***p < 0.001; ns, no significant difference; DOC, disorders of consciousness; UWS, unresponsive wakefulness syndrome; MCS, minimally conscious state; NCS-R, nociception coma scale-revised.

When we compared NCS-R total scores for MCS patients between conditions, there were statistically significant differences in NCS-R total scores between the three conditions (H = 14.93, *p* < 0.001), including a significant difference between physiotherapy and baseline (*p* < 0.001), a significant difference between noxious stimuli and baseline (*p* < 0.001), and a significant difference between noxious stimuli and physiotherapy (*p* < 0.001) (Figure 1).

When we compared NCS-R total scores for UWS patients between conditions, there was a statistically significant difference in NCS-R total scores between the three conditions (H = 76.80, *p* < 0.001), including a statistical difference between baseline and physiotherapy (*p* < 0.05), a significant difference between noxious stimuli and baseline (*p* < 0.001), and a significant difference between noxious stimuli and physiotherapy (*p* < 0.001) (Figure 1).

When analyzing the effect of the consciousness on NCS-R scores, there was no statistical difference between MCS and UWS at baseline (U = 378, z = –1.35, *p* = 0.178). However, there was a statistically significant difference between MCS and UWS during physiotherapy (U = 1,362, z = –3.06, *p* < 0.01) and under noxious stimuli (U = 5142.5, z = –11.22, *p* < 0.001) (Figure 1).

## Discussion

Our study examined the pain response of MCS and UWS patients under different conditions (e.g., baseline/non-noxious, physiotherapy/potential noxious stimulus, and noxious stimulus). There was a statistically significant difference in NCS-R total scores between the three conditions for DOC patients. In addition, this study found that NCS-R total scores during physiotherapy were significantly higher in MCS patients than at baseline, and scores during physiotherapy were also higher in UWS patients than at baseline. In addition, MCS patients had significantly higher total NCS-R values during physiotherapy and noxious stimulus than UWS patients.

Previous studies have shown that the NCS-R is sensitive to assessing patients’ responsiveness to noxious stimuli (Chatelle et al., 2012, 2018; Vink et al., 2014). As more researchers focus on pain management in DOC patients, timely detection of potential pain signs has become an important clinical research topic. In a previous study, it was found that patients responded better to personalized stimuli than to standard stimuli (Formisano et al., 2020). In addition to the above stimuli, other stimuli have been used to elicit pain responses in patients, including bilateral nipple pinching, acupuncture at the root of the nose, and electrical stimulation of the median nerve (Tsetsou et al., 2015). According to a previous study, repeated noxious stimuli or persistent painful conditions should be avoided and/or treated at DOC to prevent the long-term effects mentioned above. Repeated noxious stimuli may also cause implicit memory traces, hyperalgesia, uncontrolled anxiety, and behavioral changes (Garcia-Larrea and Bastuji, 2018). All of this suggests that appropriate sensory stimulation is critical to pain diagnosis and treatment.

In the present study, it was found that most DOC patients did not show significant pain responses when they did not receive stimuli (at baseline). Responses elicited by physiotherapy and noxious stimuli were significantly higher than baseline. In addition, the responses elicited by noxious stimulus were significantly greater than those elicited by physiotherapy. According to the updated NCS-R research, a total score of two or more is associated with nociception (Chatelle et al., 2012) and five is associated with potential pain (Bonin et al., 2020). In the present study, most MCS patients had NCS-R scores above four, some UWS patients had scores above four, and all patients’ scores were below four during physiotherapy. This indicates that the physical therapy used in this study resulted in less pain in the patients. The mean disease progression here was 4 months, with a mean of 5.24 months. Patients in the chronic stage were more likely to have spasticity, ossification, and the other causes mentioned above (Barr et al., 2013; Thibaut et al., 2015; Zasler et al., 2021, 2022), which may partly explain why they performed worse with physiotherapy than in previous studies (Bonin et al., 2022). This could also be related to limb range of motion, frequency of physiotherapy, and other therapy related factors. The lack of objective behavioral tools to assess movement function could also be a factor.

The goal of manual therapy was to modulate and relieve pain. This could even be related to the analgesic effect of physiotherapy in DOC patients. Then a common clinical question arises: what is the purpose of using noxious stimuli in DOC patients? For conscious diagnosis or for conscious therapy? Pain is influenced by both peripheral and central mechanisms. Due to various pathological mechanisms, chronic pain often has secondary consequences such as anxiety and depression. This can impact the recovery of DOC patients suffering from chronic pain. In addition, patients may even experience negative side effects as a result of improper withdrawal or inappropriate use of pain medications. The two case reports mentioned above (Lanzillo et al., 2016; Formisano et al., 2019) shown that, especially in MCS patients, we need to pay attention to pain during physiotherapy and investigate the causes of pain responses (the source of sensory noxious stimulus) to promote the recovery of consciousness and movement function.

In our study, a significant difference was found between MCS and UWS during physiotherapy and under noxious stimuli. This suggests that the patient’s state of consciousness is partly responsible for the pain response. A recent study using neuroimaging (^18^F-FDG PET) showed that an NCS-R threshold of five can be used as a warning signal to identify potentially painful patients with covert consciousness during stimulation or physiotherapy (Bonin et al., 2020). Based on the scores of the subjects in the present study, we found that patients’ scores were below five during physiotherapy (i.e., manual therapy), implying that this therapy may be more likely to improve patients’ reactivity, but to elicit potentially noxious stimuli such as disease development. In addition, four UWS patients scored higher than five on the noxious stimuli. According to recent research, these patients could be diagnosed with MCS star with covert consciousness (diagnosed as UWS based on CRS-R assessment but with minimal preservation of brain metabolism) (Thibaut et al., 2021) or cognitive motor dissociation (CMD, patients show no detectable command-following behaviors, but there is clear evidence of command-following brain activity) (Egbebike et al., 2022). One research study found that three out of seven DOC patients could communicate using the asynchronous brain-computer interface (BCI), suggesting that BCI can be a helpful tool for bedside communication with DOC patients (Huang et al., 2021). In addition, another study found that 83.33% of patients with CMD diagnosed with BCI were able to regain consciousness, indicating that clinicians can recognize the awareness of BCI-based clinical diagnosis in DOC patients (Pan et al., 2020). Similarly, patients with DOC cannot communicate subjective pain sensations, and BCI tools maybe used to help clinicians or researchers identify signs of pain.

## Study limitations

A limitation of this study is that there was no objective assessment of patients spasticity using a standard instrument that can more accurately diagnose the severity of patients’ spasticity (Thibaut et al., 2015). Therefore, further studies with objective spasticity instruments are needed to validate the preliminary results. In addition, in the current study, all patients were diagnosed using a repetitive CRS-R scale, which may result in some patients with MCS star being misdiagnosed as UWS. In the future, multimodal neuroimaging and BCI methods will be used to diagnose patients’ consciousness to obtain an accurate result of pain responses during physiotherapy. Finally, we have not investigated the relationship between physical therapy and recovery of consciousness, which we will do in future studies.

## Conclusion

In conclusion, our study found that physiotherapy improved activity responsiveness and caused less potential pain in DOC patients, whereas noxious stimuli elicited pain perception, especially in MCS patients. Consequently, physiotherapy (manual therapy) may be suitable to restore motor function and even cognition in DOC patients while reducing pain perception.

## Data availability statement

The raw data supporting the conclusions of this article will be made available by the authors, without undue reservation.

## Ethics statement

The studies involving human participants were reviewed and approved by the Ethics Committee of Shanghai Yongci Rehabilitation Hospital. The patients/participants provided their written informed consent to participate in this study.

## Author contributions

JS, ST, BY, DX, and TF collected the data and managed the patients. LC and GL substantially contributed to analysis of data. JS and GL substantially contributed to interpretation of data. JS substantially contributed to study supervision. All authors contributed to the article and approved the submitted version.
