# Supplementary material for: Pain assessment during physiotherapy and noxious stimuli in patients with disorders of consciousness: A preliminary study
Source: Front Integr Neurosci. 2022 Sep 8;16:962077. doi: 10.3389/fnint.2022.962077 (PMC9492971; doi:10.3389/fnint.2022.962077)
Supplement: Supplementary file 1 [file Table_1.DOCX]

**SUPPLEMENTARY TABLE |** The details of NCS-R scores of all DOC patients (n = 93)

| ID | Diagnosis | CRS-R scores | NCS-R scores | | |
| --- | --- | --- | --- | --- | --- |
|  |  |  | Baseline | Physiotherapy | Noxious |
| P1 | UWS | 5 | 1 | 1 | 3 |
| P2 | UWS | 10 | 1 | 1 | **5** |
| P3 | UWS | 7 | 0 | 1 | 3 |
| P4 | UWS | 4 | 1 | 1 | 3 |
| P5 | UWS | 8 | 1 | 1 | 3 |
| P6 | UWS | 3 | 0 | 1 | 2 |
| P7 | UWS | 8 | 0 | 1 | 4 |
| P8 | UWS | 5 | 0 | 1 | **5** |
| P9 | UWS | 4 | 1 | 1 | 4 |
| P10 | UWS | 5 | 0 | 1 | 3 |
| P11 | UWS | 4 | 1 | 1 | 3 |
| P12 | UWS | 5 | 0 | 1 | 4 |
| P13 | UWS | 4 | 0 | **2** | 4 |
| P14 | UWS | 5 | 0 | 1 | 3 |
| P15 | UWS | 4 | 1 | 1 | 3 |
| P16 | UWS | 7 | 1 | 1 | 4 |
| P17 | UWS | 5 | 1 | 1 | 4 |
| P18 | UWS | 5 | 0 | **2** | 4 |
| P19 | UWS | 5 | 0 | **2** | **5** |
| P20 | UWS | 5 | 1 | 1 | 3 |
| P21 | MCS- | 8 | 1 | 1 | 4 |
| P22 | MCS- | 10 | 1 | 1 | **5** |
| P23 | UWS | 10 | 0 | 1 | 3 |
| P24 | MCS+ | 12 | 1 | 1 | 5 |
| P25 | MCS- | 10 | 1 | 1 | 4 |
| P26 | MCS- | 15 | 0 | **2** | **8** |
| P27 | MCS- | 13 | 1 | **3** | **7** |
| P28 | UWS | 7 | 0 | 1 | 3 |
| P29 | UWS | 4 | 1 | 1 | 3 |
| P30 | MCS- | 10 | 0 | 1 | **5** |
| P31 | MCS- | 14 | 1 | **2** | **7** |
| P32 | UWS | 5 | 0 | 1 | 3 |
| P33 | MCS- | 9 | 1 | 1 | **5** |
| P34 | UWS | 8 | 1 | 1 | 4 |
| P35 | UWS | 7 | 0 | **2** | 4 |
| P36 | UWS | 9 | 1 | 1 | 4 |
| P37 | MCS | 9 | 1 | 1 | **5** |
| P38 | UWS | 5 | 1 | 1 | 3 |
| P39 | UWS | 8 | **2** | **2** | 3 |
| P40 | MCS | 11 | 1 | 1 | **6** |
| P41 | UWS | 4 | 1 | 1 | 4 |
| P42 | UWS | 9 | 1 | 1 | 4 |
| P43 | MCS- | 9 | **2** | **2** | **5** |
| P44 | UWS | 5 | 1 | 1 | 4 |
| P45 | MCS- | 10 | 1 | 1 | 3 |
| P46 | MCS- | 8 | 0 | 1 | 3 |
| P47 | MCS+ | 15 | 0 | **2** | **7** |
| P48 | MCS- | 10 | 1 | 1 | 3 |
| P49 | MCS- | 6 | 0 | 1 | **5** |
| P50 | MCS- | 8 | 1 | 1 | 3 |
| P51 | MCS- | 6 | 0 | 1 | **5** |
| P52 | MCS- | 9 | 0 | **2** | **6** |
| P53 | MCS+ | 13 | 1 | 1 | 4 |
| P54 | MCS- | 8 | 0 | **2** | **5** |
| P55 | MCS- | 9 | 0 | 1 | **5** |
| P56 | MCS- | 9 | 1 | **2** | 4 |
| P57 | MCS- | 9 | 1 | **2** | 4 |
| P58 | MCS- | 9 | 1 | 1 | 4 |
| P59 | MCS- | 9 | 0 | 1 | 4 |
| P60 | MCS- | 15 | 0 | 1 | **7** |
| P61 | MCS- | 12 | 1 | 3 | **6** |
| P62 | MCS- | 13 | 1 | **2** | **7** |
| P63 | MCS- | 6 | 1 | **2** | 3 |
| P64 | MCS- | 15 | 1 | 1 | **6** |
| P65 | MCS | 16 | 1 | 1 | **7** |
| P66 | MCS- | 13 | 1 | **3** | **7** |
| P67 | MCS | 8 | 1 | 1 | 4 |
| P68 | MCS | 9 | **2** | **2** | 3 |
| P69 | UWS | 6 | **2** | **2** | 3 |
| P70 | UWS | 7 | 1 | 1 | 4 |
| P71 | MCS- | 11 | 1 | **2** | **5** |
| P72 | UWS | 8 | 0 | **2** | 4 |
| P73 | UWS | 5 | **2** | **2** | 4 |
| P74 | UWS | 8 | 1 | **2** | 4 |
| P75 | MCS- | 7 | 0 | **2** | 4 |
| P76 | MCS- | 10 | 0 | **3** | **5** |
| P77 | MCS- | 9 | 1 | **2** | **5** |
| P78 | UWS | 6 | 0 | **2** | 4 |
| P79 | UWS | 10 | 0 | 1 | 4 |
| P80 | UWS | 10 | 1 | 1 | 4 |
| P81 | MCS- | 12 | 0 | 1 | 4 |
| P82 | MCS- | 9 | 0 | 1 | 4 |
| P83 | UWS | 5 | 0 | 1 | 3 |
| P84 | UWS | 5 | 1 | **2** | 3 |
| P85 | MCS+ | 14 | 0 | **3** | **5** |
| P86 | MCS+ | 16 | 0 | **2** | **6** |
| P87 | MCS- | 14 | 1 | **3** | **6** |
| P88 | MCS- | 13 | 1 | **2** | **5** |
| P89 | UWS | 6 | 1 | 1 | 4 |
| P90 | UWS | 11 | 1 | 1 | **6** |
| P91 | MCS- | 11 | **2** | **2** | **7** |
| P92 | MCS- | 9 | 1 | 1 | 4 |
| P93 | MCS- | 10 | 0 | **3** | **5** |

P, patient; DOC: disorders of consciousness; UWS: unresponsive wakefulness syndrome, MCS: minimally conscious state; CRS-R: Coma Recovery Scale-Revised; NCS-R: Nociception Coma Scale-Revised
